# Advanced Electrochemiluminescence DNA Aptasensor for Sensitive Detection of Cancer Biomarkers

**DOI:** 10.34172/apb.47032

**Published:** 2026-05-24

**Authors:** Golnoush Rahvar, Mahdis Ojaghlou, Kimia Rostami, Mansour Mahmoudpour, Zahra Karimzadeh

**Affiliations:** ^1^Research Center for Pharmaceutical Nanotechnology, Biomedicine Institute, Tabriz University of Medical Sciences, Tabriz, Iran; ^2^Miandoab Faculty of Medical Sciences, Urmia University of Medical Sciences, Urmia, Iran; ^3^Engineered Biomaterials Research Center, Khazar University, Baku, Azerbaijan

**Keywords:** Electrochemiluminescence, DNA aptasensor, Nanomaterials, Cancer biomarkers

## Abstract

Cancer continues to be the most important global health issue, requiring effective monitoring for improved diagnosis and survival. By combining the molecular recognition capabilities of aptamers with the signal enhancement features of electrochemiluminescence (ECL), DNA-based ECL aptasensors have emerged as a highly specific and sensitive method for determination of cancer biomarkers. This review critically evaluates recent advances in ECL aptasensor design, signal amplification strategies, aptamer design, and clinical applications. Nanomaterials and aptamer configurations are discussed in terms of their roles in improving ECL performance and detection capability. Key challenges, including reproducibility, stability in complex matrices, and point-of-care applicability, are emphasized. Finally, emerging strategies, including advanced nanomaterials, integrated platforms, and computational approaches, are briefly outlined to guide future development of robust and portable ECL aptasensors for cancer diagnostics.

## Introduction

 Cancer is the second leading contributor to global mortality, responsible for roughly one out of every six deaths.^1^ Conventional imaging sensory systems and laboratory-based biomarker assays (including enzyme-linked immunosorbent assay (ELISA) and polymerase chain reaction (PCR)), while clinically valuable, often suffer from inadequate sensitivity for early-stage detection, high operational costs, and slow analytical turnaround.^2,3^ Accordingly, it is necessary to develop selective and sensitive sensors with greater utility to detect different biomarkers in all types of cancers.^4^

 The electrochemiluminescence (ECL) method has attained considerable attention in the determination of various cancer biomarkers owing to its selectivity towards different targets, wide dynamic range, and ability to integrate with portable devices.^5^ This approach has gained considerable attention in biosensory systems due to its excellent sensitivity, its independence from external light sources, and its broad detection range.^6^ To improve the efficiency of ECL-based sensors, aptamers-short, single-stranded RNA or DNA oligonucleotides possessing strong affinity and selectivity for target molecules-have been extensively employed as molecular recognition components.^7^ Aptamers enable rapid, efficient, and robust identification of biomolecules relevant to medical diagnostics, food safety analysis, and environmental monitoring.^8^ Nanoparticles and nanomaterials further enhance ECL aptasensors by providing improved electron transfer, and high surface area. enabling efficient signal amplification and stable aptamer immobilization. Their integration significantly improves sensitivity and lowers limit of detection (LOD) for cancer biomarkers.^9,10^ Incorporating ECL into aptasensor platforms has resulted in the construction of powerful analytical tools that combine the advantages of both ECL and aptamers. Despite extensive studies on individual materials and biomarkers, there remains a lack of systematic evaluation of which combinations of nanomaterials, co-reactants, and aptamer designs provide optimal performance, especially for multiplexed and clinically relevant applications.

 This review first considers the basic principles of ECL-based aptasensors and the mechanistic and functional classification of ECL aptasensors, highlighting strategies for signal amplification, aptamer configuration, and translational device design. Then, key challenges and emerging trends are discussed to establish the next generation of cancer diagnostic systems.

## Electrochemiluminescence (ECL) based aptasensors

 ECL is a fascinating phenomenon that merges aspects of electrochemistry and luminescence for extremely sensitive determination of analytes. In ECL-based assays, an electrochemical reaction induces excited states in the species that produce light after returning to the ground state. ECL-based aptasensors represent a specific class of biosensors that employ aptamers as recognition tools. Aptasensors have gained prominence due to the versatility of aptamers, which may be designed to bind to numerous targets, including small molecules, proteins, and entire cells.^11,12^ ECL aptasensors operate in diverse modes, including signal-on sensors, where target binding increases ECL intensity, signal-off sensors, where target binding quenches ECL emission, and ratiometric sensors, employing dual luminophores for improved accuracy.

## Strategies for signal enhancement

 Signal amplification is fundamental to achieve ultra-sensitive performance in ECL aptasensing. Modern amplification strategies can be classified into three major functional groups: nanomaterial-mediated enhancement,^7^ co-reactant/luminophore engineering,^13^ and enzymatic or catalytic amplification. These approaches improve signal intensity, stability, and specificity, especially when integrated with aptamer-based molecular recognition.

###  Nanomaterials for signal amplification

 Nanomaterials continue to serve as the core amplification elements of ECL aptasensors. Nanomaterials are nanosized structures or components, typically between 1 and 100 nm.^14,15^ Due to their excellent features at this scale, including exceptional physical and chemical features, nanomaterials increase the accuracy and sensitivity of biosensors by increasing the contact surface between the analyte and the sensors.^16^ To date, various types of nanomaterials, containing metal nanoparticles, carbon-based nanomaterials (such as carbon nanotubes and graphene), semiconductor NPs (such as quantum dots (QDs)), novel organic nanomaterials, *etc.,* have been investigated for use in ECL aptasensors.^17^ Nanomaterials function as aptamer carriers, electrode modifiers, catalysts, co-reactants, or even luminophores within ECL systems.

####  Metal nanomaterials

 Metal nanoparticles in ECL aptasensors are mainly applied to increase sensitivity, signal amplification, and improve electron transport. Most of the metal nanoparticles used in ECL aptasensors are compatible with all kinds of biomolecules and possess good compatibility. Gold (Au), silver (Ag), platinum (Pt), and palladium (Pd) are common metals used for this purpose.^18^

 AuNPs are particularly popular as signal amplification tags, owing to their facile synthesis, chemical stability, high biocompatibility, and strong Au–S bonding with thiolated aptamers. AuNPs enhance ECL signals by acting simultaneously as electrode modifiers, catalytic centers, energy acceptors, and nanocarriers for luminophores.^19^ For example, Figure 1A (I) shows that AuNPs as signal amplification tags could enhance the performance of ECL biosensors through electrode modification, energy acceptor, and signal enhancer.^20^

**Figure 1:**
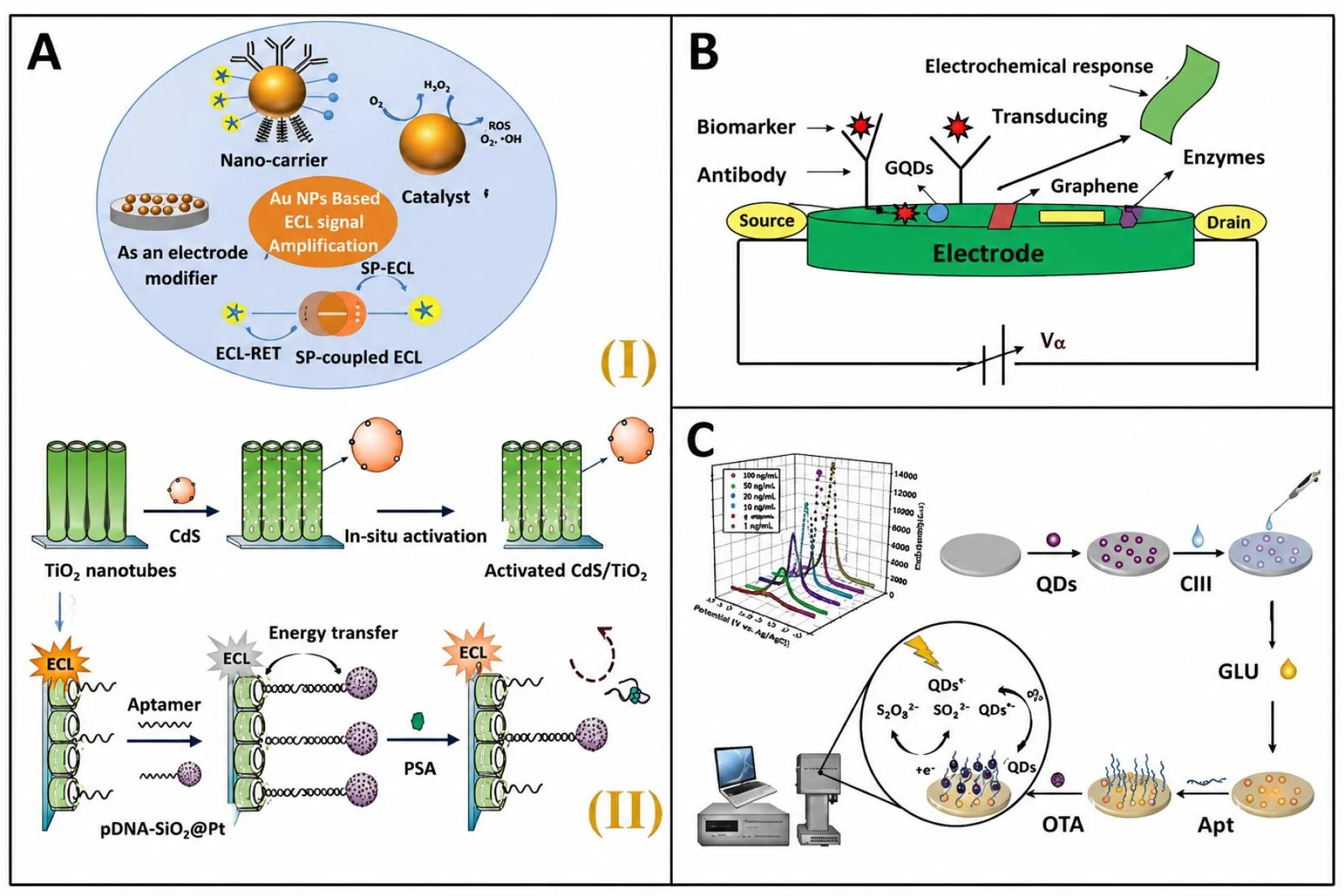
Types of nanomaterials used in ECL aptasensors, (A) metal nanomaterials, (I): Au NPs as nano-carriers, electrode modifiers, and catalysts. Reprinted with permission from Ref.20 (II) CdS nanocrystals used as enhanced emitters in ECL. Reprinted with permission from Ref.^22^; (B) Carbon-based nanomaterial, graphene in ECL biosensing. Reprinted with permission from Ref^23^; (C) Quantum dots (QDs), use of CdSe@CdS QDs for specific and sensitive detection. Reprinted with permission from Ref.^24^

 In addition, Lin et al designed a platform using bio-encoded AuNPs and DNAzyme sequence for dual amplification in ECL signal.^21^ The proposed system revealed exceptional sensitivity with LOD of 0.2 pmol.L^-1^ for thrombin detection and demonstrated good performance in real samples analysis.

 Silver nanoparticles (AgNPs) are broadly used in biomedical research on account of their quantum size-dependent plasmonic features, low toxicity, high biocompatibility, and small grain diameter. Of the cases taken, the most reported about the use of AgNPs in ECL aptasensing is because of their use in the power supply. Studies have shown that compared to AuNPs, AgNPs exhibit enhanced electron transfer activity, enabling them to promote the movement of electrons. Additionally, various biomolecules can be attached to AgNPs via chemical interactions. A research conducted by Cheng et al. demonstrated that the silver-amine coupling method enhanced the intensity and sensitivity of ECL for kanamycin detection while reducing the signal response time.^19,25^ In some cases, a combination of different nanometals is used to maximize the efficiency of ECL aptasensors exhibiting higher synthetic complexity related to their monometallic counterparts. For example, according to the results shown in the Figure 1A (II), CdS/TiO_2_ nanotubes were used as a highly effective ECL emitter in PSA detection with a 265-fold increase in ECL signal.^22^ Here, the SiO_2_@Pt NPs served as ECL quenchers for the CdS/TiO_2_ NTs. The quantification of PSA was amplified by ECL signals, with linearity of 0.001 to 50 ng mL^-1^ and a low LOD of 0.4 pg mL^-1^.

####  Carbon-based nanomaterials

 Carbon-based nanomaterials are essential for amplifying ECL signals. Compared to traditional carbon materials, graphene and carbon nanotubes (CNTs) provide superior electrical conductivity, mechanical strength, and surface functionality.^26^ The π-π stacking on carbon nanotube surfaces promotes the simple adsorption of nucleic acids. Single/multi-walled carbon nanotubes (SWCNTs/MWCNTs) are the two categories into which CNTs fall.^23,27^ Graphene is characterized by its two-dimensional structure and atomic-scale thickness of a single carbon atom layer. Therefore, electrodes modified with graphene illustrate a good analytical response. The powerful interaction between graphene and single-stranded nucleic acids has been utilized to produce effective and simple ECL aptasensors, and covalent modification on graphene have been used for aptamer immobilization, as demonstrated in Figure 1B. The main benefit of graphene over other carbon-based nanomaterials is its large-scale and low-cost manufacturing ability in the establishment of biosensors. Graphene’s great surface area and low thickness provide high electrical/thermal conductivity compared to CNTs.^26,27^ The incorporation of metal nanoparticles with graphene is commonly used to improve biosensing properties. In short, the smart and appropriate integration of NMs in aptasensors increases their sensitivity, selectivity, output current, and resistance to electrode deposition.^28^

####  Quantum dots (QDs)

 Quantum dots are semiconductor nanocrystals that serve as high-efficiency ECL luminophores and signal amplification agents. Their size-dependent emission at multiple desired frequencies, high photostability, broad excitation spectra, and resistance to photobleaching make them ideal for repeated ECL cycles. QDs play a dual role as energy donors and acceptors in resonance energy transfer (RET)-based mechanisms, enabling ratiometric and multiplexed ECL sensing.^29^ Additionally, QDs serve effectively as both acceptors and donors in luminescent RET. With the aid of NH₂ and COOH functional groups, they are capable of binding a greater quantity of aptamers. These advantages have made them an efficient tool in medical diagnostics.^30,31^ In a study by Du et al., nitrogen-doped graphene QDs embedded in silica (NGQDs@SiO₂) nanocomposites were employed to fabricate a well-developed ECL aptasensor for the determination of thrombin. The developed ECL aptasensor exhibited remarkable performance, enabling the determination of thrombin with a LOD of 23.1 fM over a concentration range of 2.0 pM to 50 nM.^32^ Similarly, a study conducted by Jia and coworkers, a label-free ECL aptasensor based on CdSe@CdS QDs was fabricated for the specific and sensitive quantification of ochratoxin A (OTA). As revealed in Figure 1C, abundant QDs were immobilized on the Au electrode via chitosan. The outcomes showed that the CdSe@CdS quantum dots possess excellent stability and enhance the ECL intensity and accelerate the reactions in the electrolyte.^24^

####  MOFs

 MOFs represent powerful signal amplification platforms due to their high porosity and tunable electrochemical properties.^33^ Their well-defined crystalline structures allow efficient loading of luminophores, co-reactants, and aptamers. Recent advances include the incorporation of Ru(bpy)₃^2+^, luminol, and QDs into MOF architectures to construct highly active ECL frameworks. For example, Zn tetrakis(carboxyphenyl)-porphyrin (TCPP) linkers within MOF-525 showed excellent ECL performance to assess protein kinase A activity (Figure 2A).^34^ Utilizing MOF-525-Zn as signal amplification probes, a potential ECL sensor was established, exhibiting linearity in the range of 0.01 to 20 U mL^−1^ with LOD of 0.005 U mL^−1^. Additionally, According to the Figure 2B an ECL biosensor was developed via triethanolamine-modified AuNPs (TEOA@AuNPs) as both a nanocarrier and a co-reactant for carcinoembryonic antigen (CEA) aptamers, along with CdS QDs integrated into a metal–organic framework (CdS QDs@MOF) as dual co-reactants within the Ru(bpy)₃^2+^-based ECL system.^35^ Upon exposure to a CEA solution, a low LOD of 8.5 × 10⁻⁵ ng·mL⁻¹ was achieved in linear CEA concentration.

**Figure 2:**
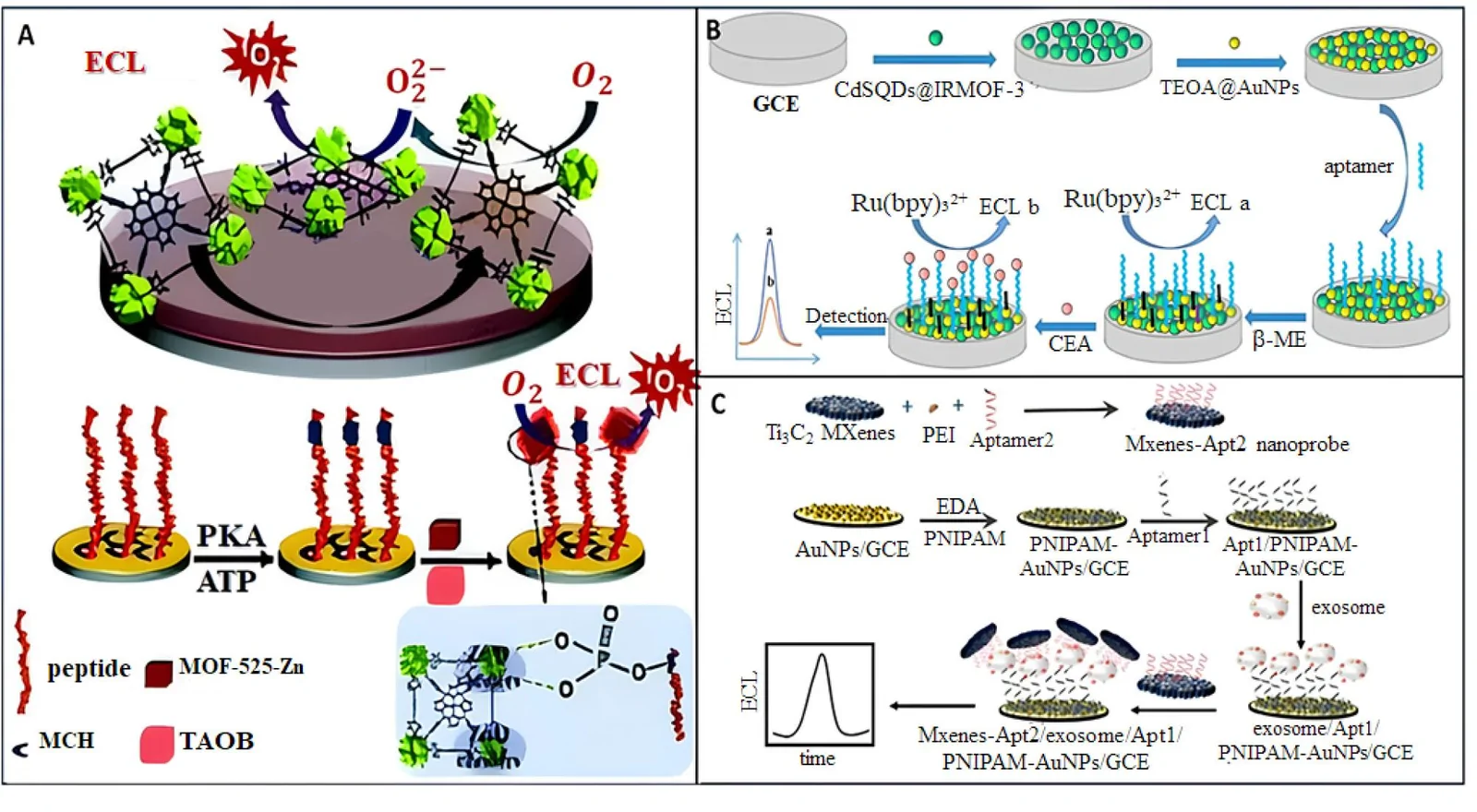
(A) An example of the ECL modulation mechanism and the ECL sensing system made up of Zr-based MOF. Reprinted with permission from Ref.^34^ (B) The process of CEA quantification using triethanolamine modified on AuNPs and CdS QDs@MOF in a label-free ECL. Reprinted with permission from Ref.^35^; (C) Schematic illustration of the use of Ti3C2 MXenes nanosheets in ECL aptamers for the identification and analysis of exosome activity. Reprinted with permission from Ref.^36^

####  MXene

 MXenes are emerging two-dimensional nanomaterials that exhibit exceptional conductivity and catalytic activity, making them highly effective signal enhancers in ECL aptasensors.^37-39^ Among various MXenes, Ti_3_C_2_ MXenes have shown significant capability in many applications, including catalysis, pollutant processing, and biosensing. Their broad surface area enables efficient loading of biomolecules and enhances electrochemical sensing signals. In Zhang et al. study, a platform for exosome quantification was created utilizing aptamer-modified Ti_3_C_2_ MXenes nanosheets as ECL nanoprobes.^36^ As shown in Figure 2C, the unique properties of Ti_3_C_2_ MXenes facilitate the effective adsorption of exosomes via the aptamer recognizing the EpCAM protein. This setting significantly improved the luminol ECL signals, resulting in an efficient sensor for MCF-7 exosomes recognition in the serum, with a LOD of 125 particles μL^-1^, and could become a valuable tool for exosome determination in clinical evaluations.

###  Co-Reactants and Luminophores

 Co-reactants, including tripropylamine (TPA) and persulfate (S₂O₈^2-^), participate in redox reactions with luminophores, producing reactive intermediates that amplify the ECL signal. Proper selection of luminophores and co-reactants maximizes signal intensity while minimizing background interference. Traditional Ru(bpy)₃^2+^ complexes are still widely used on account of their stability and high quantum yield. Modified Ru complexes, nanomaterial-linked luminophores, and self-enhanced systems further improve stability and signal intensity. For example, in a study conducted by Fang and colleagues, a dual-mode biosensor based on ECL and photothermal effects was designed to detect exosomes using materials such as MXenes as signal enhancers and black phosphorus QDs (BPQDs).^40^ In this work, BPQDs were introduced as a catalyst for the oxidation of Ru(dcbpy)_3_^2+^, and the self-enhancing ECL system Ru(dcbpy)_3_^2+^@BPQDs was able to produce a strong ECL signal, which also exhibited features like reduced energy dissipation and reduced electron transfer distance. To further improve the ECL signal and increase the quantity of immobilized Ru(dcbpy)_3_^2+^ and BPQD, MXenes were also used as supports.

 In another study conducted by Zhang et al., [Ru (dcbpy)_3_]^2+^ functionalized 2D/3D hybrid metal-organic frameworks (Ru-BTC-MOFs) were produced. This process utilized Ru(dcbpy)₃Cl₂, and H₃BTC as the organic ligand and Zn(NO₃)₂ as the metal ion source. The researchers thoroughly investigated how various parameters, such as electrodeposition voltage, time, and concentrations of H₃BTC and Ru(dcbpy)₃Cl₂, influenced the ECL performance and morphology of the Ru-BTC-MOFs. To create a potent ECL biosensor for CEA detection, the researchers modified the Ru-BTC-MOFs with AuNPs and aptamers. The electron conduction between the Ru-BTC-MOFs and tri-n-propylamine (TPA) is inhibited through binding of aptamer to CEA, thereby reducing the ECL intensity. The results exhibited a linearity for CEA concentration logarithm with the ECL intensity over the range of 10.0 fg/mL- 10.0 ng/mL with LOD of 5.56 fg/mL.^41^ These systems enhanced the signal by increasing loading density, improving electron transfer, and reducing background noise.

###  Enzymatic and catalytic amplification

 Enzyme- or nanozyme-mediated amplification further enhances ECL signals by catalyzing the generation of reactive species. Peroxidase-mimicking nanoparticles or horseradish peroxidase (HRP) catalyze luminol oxidation, resulting in strong, reproducible ECL outputs. When combined with aptamers, these enzymes guarantee that signal generation occurs exclusively in the existence of target biomarkers, thereby eliminating false positives results. Catalytic nanomaterials, such as Pt nanoparticles, MXenes, or hybrid nanocomposites, can substitute or complement enzymes for enhanced stability. In the study by Zhang et al., an exceptionally selective and sensitive ECL aptasensor was created for quantifying PDGF-BB.^42^ This innovative sensor utilized a multilayered nanocomposite film made of AuNPs and electrochemically reduced graphene (EG), which was constructed on a glassy carbon electrode (GCE) utilizing a co-electrodeposition technique. The AuNPs-EG composite provided excellent conductivity, enhanced the surface area for attaching a significant amount of the primary aptamer (Apt1), enhanced electron transfer, and improved biocompatibility, thereby enhancing the determination signal. Additionally, a signaling probe (GOD–Apt2–AuNPs) was constructed by labeling a secondary aptamer with glucose oxidase (GOD) and AuNPs. The system’s sensitivity was further enhanced by the *in-situ* production of H_2_O_2_ through the GOD-glucose reaction, coupled with the AuNPs’ catalytic properties involving luminol and H_2_O_2_. In optimum, the ECL aptasensor demonstrated impressive analytical capabilities for quantifying PDGF-BB with a LOD of 1.7 × 10⁻¹⁴ mol L⁻¹ over a concentration range of 1.0 × 10⁻¹³ to 5.0 × 10⁻¹⁰ mol L⁻¹. The recovery rates were found to be between 85.0% and 110%.

## Application in biomarker detection

 Cancer is characterized by the unregulated proliferation of cells resulting from alterations in the expression and condition of specific genes, which provide both somatic and germinal cells with survival advantages for the ability to proliferate without limits. In the context of cancer, biomarkers serve a vital function in disease detection, outcome prediction, patient monitoring, and treatment selection. Various technologies are employed to determine the tumor stage, subtype, and response to therapy. The identification of patterns in adjacent cells, as well as in more remote and readily accessible areas of the body, can also impact cancer treatment strategies. In clinical research, biomarkers can offer profound insights into the disease’s progression. Some biomarkers are specific to certain cancers or are non-specific, reflecting cellular courses contributing to cancer development. The following section reviews representative ECL aptasensing studies for determining specific and non-specific cancer biomarkers have been reviewed and summarized in Table 1.

**Table 1:** Comparative analytical performance of ECL aptasensors for representative cancer biomarkers

**Biomarker**	**Nanomaterial /** **Sensing Platform**	**Signal amplification mechanism**	**LOD**	**Dynamic Range**	**Selectivity**	**References**
MUC1	Ru(bpy)32 + @SiO2 NPs with CD63 aptamer	Luminophore amplification	NR	NR	High	^43^
MUC1/ HER2	Ru(bpy)32 + @SiO2 (Ru@SiO2) NPs	ECL-RET	NR	well-defined linear range	High	^44^
MUC1 / miRNA-21	Dual CHA with CdS: Mn QDs and AuNP assembly	Dual catalytic hairpin assembly (CHA) + ECL-RET	0.40 fg mL-1 (MUC1)	NR	Very High	^45^
MCF-7 cells	Closed bipolar electrode/luminol-H2O2 ECL	Electrocatalytic luminol–H₂O₂ ECL	~10 cells	NR	High	^46^
MCF-7 cells	AuNPs@graphene + CQDs-MUC1 probe	Surface amplification + nanocarrier loading	230 cells mL-1	5 × 102 – 2 × 107 cells mL-1	High	^47^
HER2	MoS₂ nanosheets + carbon nanodots (CNDs)	Co-reactant enhanced Ru(bpy)32 + ECL	1.84 fg mL-1	Wide linear range	High	^48^
HER2-ECD	AuNPs/g- C3N4 /PDDA + AuPt@ZIF-67 (RET system)	RET	17.3 fg mL-1	100 fg mL-1 – 100 ng mL-1	High	^49^
PSA	Fc-graphene sheets / Au-CdS assemblies	Signal switching quenching	0.38 pg mL-1	1 pg mL-1 – 25 ng mL-1	High	^50^
PSA	ZnS QDs + Ru(dcbpy)32 + gel (DNA clamp release)	DNA clamp release amplification	1.01 fg mL-1	NR	Very High	^51^
PSA	L-Pt/PAMAM NCs + CdS/MCNTs ratiometric system	Ratiometric ECL energy transfer	0.02 ng mL-1	0.05 – 200 ng mL-1	High	^52^
PSA	CdS/TiO2 semiconductor emitter system	Semi-conductivity enhancement	0.4 pg mL-1	0.001 – 50 ng mL-1	High	^22^
PSA	CdS/Chitosan/g- C3N4 nanocomposite	Ferrocene quenching	0.14 pg mL-1	1 pg mL-1 – 100 ng mL-1	High	^53^
PSA / AFP	VMSF + magnetic graphene oxide probe	Probe release quenching	9.6 pg mL-1 (PSA)	NR	High	^54^
AFP	Pt(II) nanospheres	Aggregation-Induced ECL (AIECL) Enhancement	7.3 fg mL-1	10-5 to 102 ng mL-1	High	^55^
ProGRP	Label-free Ru(bpy)2dppz2 + ECL aptasensor	Label-free DNA binding ECL	17 nM	NR	High	^56^
NAP2	Label-free Ru(bpy)₂dppz2 + ECL aptasensor	Conformation-controlled ECL switching	NR	NR	High	^57^
NSE	MXene-Au nanocomposites + laser-induced graphene electrode	Conductivity enhancement/ probe loading enhancement	0.13 pg mL-1	0.01 – 100 ng mL-1	High	^58^
CEA	CdS-graphene nanocomposite	Electron transfer enhancement	3.8 pg mL-1	NR	High	^59^
CEA	Dual electrode CdS-C / luminol-AuNP system	Dual-signal ECL detection	0.20–0.39 pg mL-1	NR	High	^60^
CEA	Au-CdS / hemin-graphene dual quenching	Dual quenching mechanism	0.28 pg mL-1	0.8 pg mL-1 – 4 ng mL-1	High	^61^
CEA	Au–LDH–C3N4 NPs	heterogeneous coupling between LDH and C3N4	0.3 pg mL-1	0.001–20 ng mL-1	High	^62^
PDGF-BB	ABEI-functionalized AuNP nanoprobes	Luminophore amplification	NR	NR	High	^63^
PDGF-BB	CdS QDs-PAMAM / MWCNT-chitosan electrode	Sandwich signal amplification	0.13 pM	0.5 pM – 1 nM	High	^64^
PDGF-BB	β-CD/g-C3N4 composite platform	Photoinduced electron transfer	2.6 × 10-13 g mL-1	NR	High	^65^
CA125	TDN + TMSD + AuNP/Ru/ZIF probe	TMSD recycling amplification	6 × 10-3 pg mL-1	NR	Very High	^66^
Thrombin	PTG-AuNP sandwich ECL aptasensor	Ru(bpy)32 + nanocomposite amplification	1 fM	2 fM – 2 pM	Very High	^67^
Thrombin	GO-based label-free ECL aptasensor	Probe release quenching	0.40 pM	0.90 – 226 pM	High	^68^
Thrombin	QD-based sandwich ECL biosensor	QD emission amplification	NR	0 – 20 µg mL-1	High	^69^

* NR: not reported *RET: ECL resonance energy transfer *TMSD: toe-mediated strand displacement

###  Specific cancer biomarkers

####  Mucin 1 (MUC1) 

 MUC1 is a glycoprotein found on the epithelial cells surface including breast cells. In breast cancer, the MUC1 biomarker is often overexpressed up to 100-fold or aberrantly glycosylated. Its altered glycosylation can prevent immune cells from effectively binding to cancer cells and identifying them. Due to the unique features of this protein, MUC1 is being explored as a potential biomarker for the early detection of breast cancer.^70-72^ In a study by Li et al., MUC1 protein expression was detected by an ECL aptasensor on breast cancer cells ^43^. To capture the exosomes and cells, CD63 aptamer were attached on the electrode surface. MUC1-modified Ru(bpy)_3_^2+^@SiO_2_ NPs (Ru@SiO_2_ NPs) were used to increase the ECL signal. The specific MUC1 protein recognition was performed on breast cancer cell lines, and the quantitative determination was performed depending on the ECL signals. Also, another ECL aptasensing platform was formed for the concurrent discovery of miRNA-21 and MUC1 biomarkers, utilizing a dual-CHA approach.^45^ The primary cycle of the method is started by miRNA-21, which leads to a reaction due to the presentation of CdS: Mn QDs on the terminal, with LOD of 11 aM. The second cycle is activated by the MUC1-aptamer complex, resulting in the accumulation of AuNPs near the CdS: Mn QDs, causing a diminished ECL signal owing to energy transfer effects. This permits the determination of MUC1 with an LOD of 0.40 fg mL^-1^. Also, an ECL aptasensor resolved by potential was engineered for the quantification of the HER2 and MUC1 proteins presented on exosomes surface.^44^ This sensing platform enabled the selective capture of exosomes using CD63 aptamers. Two nanostructured ECL probes were employed for dual-target recognition: Ru(bpy)^2+^_3_@SiO_2_(Ru@SiO_2_) nanoparticles functionalized with a MUC1-specific aptamer, and luminol@Au (lum@Au) nanoparticles modified with a HER2-specific aptamer. These nanoprobes were positioned on two spatially separated regions of the ITO electrode, ensuring physical separation that effectively suppressed ECL-RET between them. Under optimized operational conditions, the aptasensor demonstrated well-defined linear response behaviors for both MUC1 and HER2 across four distinct exosome subtypes. The platform exhibited excellent analytical stability and reproducibility, and the platform was effectively employed for human serum analysis, indicating its promise for future clinical diagnostics.

####  MCF-7

 Another human breast cancer cell line is MCF-7, which is broadly utilized to consider estrogen receptor-positive (ER+) breast cancer. It is utilized to study the components of medication resistance, the efficacy of the medicines utilized, and to identify breast cancer.^73^ Aptamers selected to recognize MCF-7 cells can bind specifically, enabling specific quantification of breast cancer cells. For instance, Motaghi et al. created a novel bipolar electrode framework combined with the ECL method for the determination of MCF-7.^46^ To achieve specific and efficient discovery, the bipolar’s anodic pole was modified with the AS1411 aptamer. It was treated with AuNPs adjusted with a secondary aptamer. According to Figure 3A, in hydrogen peroxide (H_2_O_2_), the ECL signal from luminol was measured at the bipolar’s anode pole as the diagnostic reaction. Furthermore, 3D printed microchannels were utilized to create the bipolar anode systems to decrease the volume of tests required for testing. This aptasensor illustrates a low-cost and specific strategy with a LOD of approximately 10 cells. Also, Su and colleagues created a platform using an AuNPs@graphene complex for the sensitive and specific quantification of MCF-7 cancer cell samples.^47^ This complex was freeze-dried to modify a glassy carbon electrode and provide a high-surface matrix for capturing cancer cells with concanavalin A.

**Figure 3:**
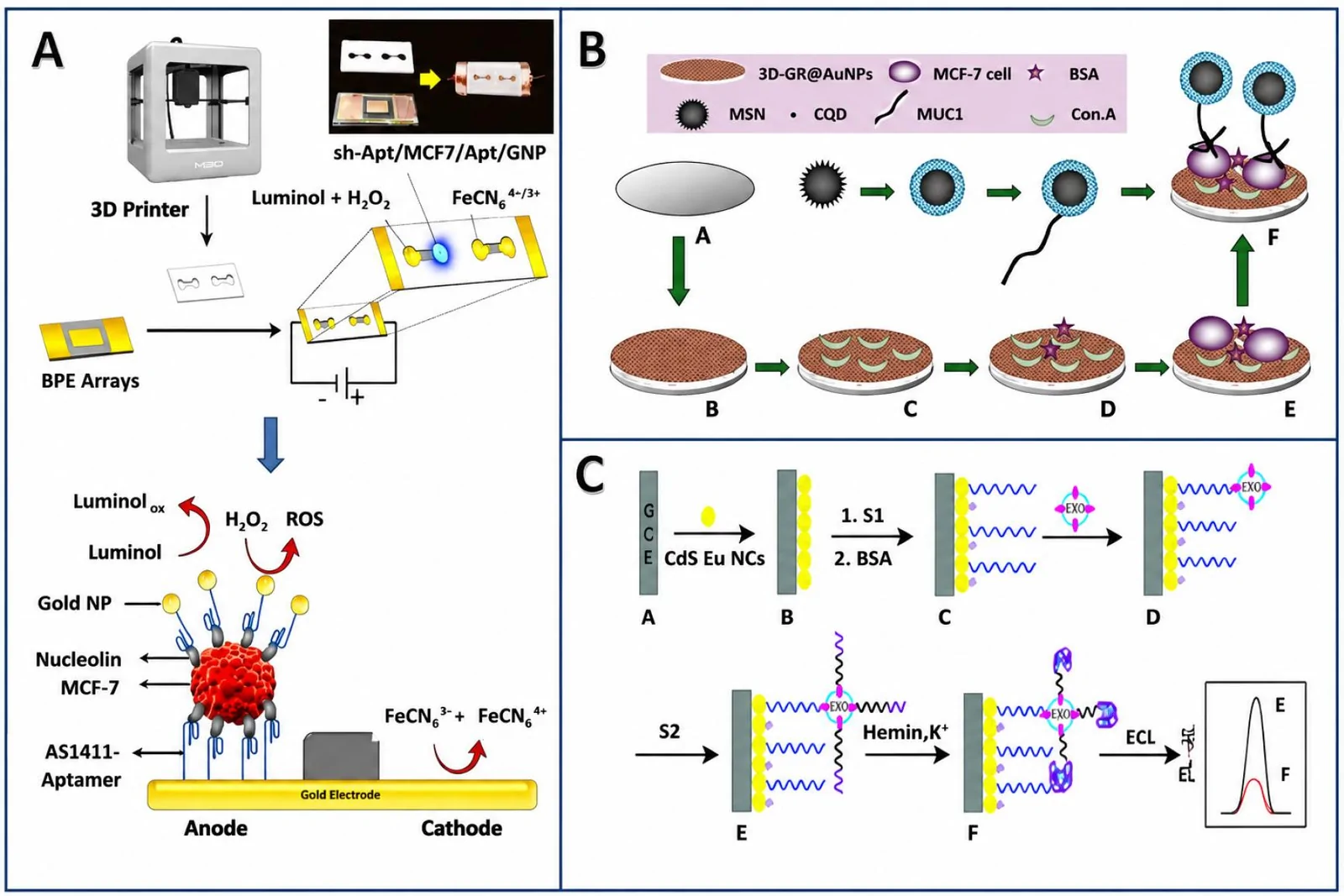
Schematic illustration of ECL-aptasensors in the MCF-7 cancer biomarkers identification, (A) The development and production of microchannels and the identification of cancer cells with ECL. Reprinted with permission from Ref.^46^ (B) The fabrication process of the ECL cyto-sensor for ultrasensitive detection of MCF-7 cancer cells on 3D-GR@AuNPs platform. Reprinted with permission from Ref.^47^ (C) Detection of MCF-7 cells by the CD63 aptamer with MPA-CdS: Eu NCs. Reprinted with permission from Ref.^74^

 According to Figure 3B, nanoparticle-coated mesoporous silica carbon quantum dots (CQDs) serve as adequate ECL detectors owing to their good biocompatibility and low cytotoxicity. These probes were conjugated to an MUC1 aptamer to bind to MUC1 on cancer cells precisely. This method showed strong capability for MCF-7 cells determination with LOD of 230 cells mL^-1^ and in the 500-2 × 10^7^ cells·mL^-1^ range. Moreover, Qiao et al. created a sensitive aptasensor for the determination of tumor-determined exosome to recognize exosomes from MCF-7 cells.^74^ Here, DNA (cytosine-5)-methyltransferase 1 (DNMT1) activity discovery and inhibitor considerations were measured via an ECL ratiometric method. Based on Figure 3C, resulting from a dual-signal ECL ratiometric test with high sensitivity, the diverse ECL changes for luminol and CdS NCs are grasped in one potential check. The method detected DNMT1 activity with LOD of 0.07 U mL^-1^ in a buffer arrangement, which can be utilized in the genuine test. For early detection and quantification of HER2 as another biomarker of breast cancer, Gutiérrez-Gálvez and colleagues developed an innovative ECL platform by combining two distinct nanomaterials: zero-dimensional carbon nanodots (CNDs) and two-dimensional molybdenum disulfide nanosheets (MoS_2_-NS).^48^ MoS_2_-NS served as a support to immobilize the SH-aptamer, which particularly binds to HER2 with high specificity. The determination of the HER2 biomarker was achieved by checking variations within the [Ru(bpy)_3_]^2+^/CND the ECL signal, which showed a low LOD of 1.84 fg mL^-1^ and a wide straight reaction extent. Besides, Zhang et al. recognized that human epidermal development calculates receptor extracellular domain (HER2-ECD) by ECL aptasensing ^49^. This investigation utilized RET between AuNPs/g-C_3_N_4_/Poly(diallyldimethylammonium chloride) (PDDA) and AuPt@ZIF-67 to identify HER2-ECD. AuNP-modified g-C_3_N_4_ acted as a signal substrate and created ECL signals in the presence of peroxydisulfate (S_2_O_8_^2–^). AuPt@ZIF-67 successfully extinguished these signals by employing ECL-RET. After a particular aptamer binding to HER2-ECD, consumption of AuPt@ZIF-67 leads to the recuperation of the ECL reaction. The aptasensor revealed a suitable linearity of 100 fg mL^-1^-100 ng mL^-1^ and a lower LOD of 17.3 fg mL^-1^.

####  Prostate-specific antigen (PSA)

 PSA is a glycoprotein commonly secreted by prostate tissue and widely used as a biomarker for the prostate cancer diagnosis. However, PSA detection in human serum can be associated with diagnostic limitations. Since it cannot distinguish between prostatitis, prostate cancer, and benign prostatic hyperplasia (BPH), it can be affected by several factors, like age and urinary tract infections, on the other hand.^16^ However, the improvement of a quick, sensitive, and determined strategy of PSA is vital for the primary diagnosis of prostate cancer. In this respect, Yang et al. introduced a platform utilizing ferrocene-graphene sheets (Fc-GNs) to eliminate ECL signal of Au-CdS flower-like assemblies.^50^ This sensor detects PSA by monitoring the signal-on state, achieving linearity from 1 pg mL⁻¹ to 25 ng mL⁻¹ with a low LOD of 0.38 pg mL⁻¹. Besides, the recoveries of 85.8–104.0% indicate extensive possible applicability in human serum. Moreover, Zhao et al. established an on/off ECL gel aptasensor employing ZnS QDs as quenchers and Cys-[Ru(dcbpy)_3_]^2+^ gel as a luminophore.^51^ This system utilizes a controlled release mechanism involving DNA clamp-like structures to induce the ZnS QDs release in the existence of PSA, leading to ECL signal enhancement. This approach demonstrated LOD of 1.01 fg mL⁻¹. Furthermore, an aptamer-based ECL test stage was created for the determination of PSA, which utilized luminol-Pt/PAMAM nanocomposites (L-Pt/PAMAM NCs) as anodic emitters and CdS nanocrystals/MCNTs as cathodic ECL emitters.^52^ According to Figure 4A, amino-modified aptamers were joined to magnetic beads and functionalized with DNA-probed L-Pt/PAMAM NCs. The magnetic L-Pt/PAMAM NCs is released in existence of PSA, by selectively binding of PSA to aptamer. At that point, the isolated NCs were hybridized with DNA adsorbed on a CdS/MCNT-coated smooth carbon cathode, diminishing the ECL signal within the cathode due to vitality exchange between emitters. As PSA concentration expanded, the anodic ECL signal from luminol expanded, whereas the cathodic signal diminished, allowing for the quantitative assurance of PSA through the ECL concentrated proportion. The developed platform demonstrated sensitive and reliable PSA quantification within the 0.05 to 200 ng mL^-1^ range, with LOD of 0.02 ng mL^-1^.

**Figure 4:**
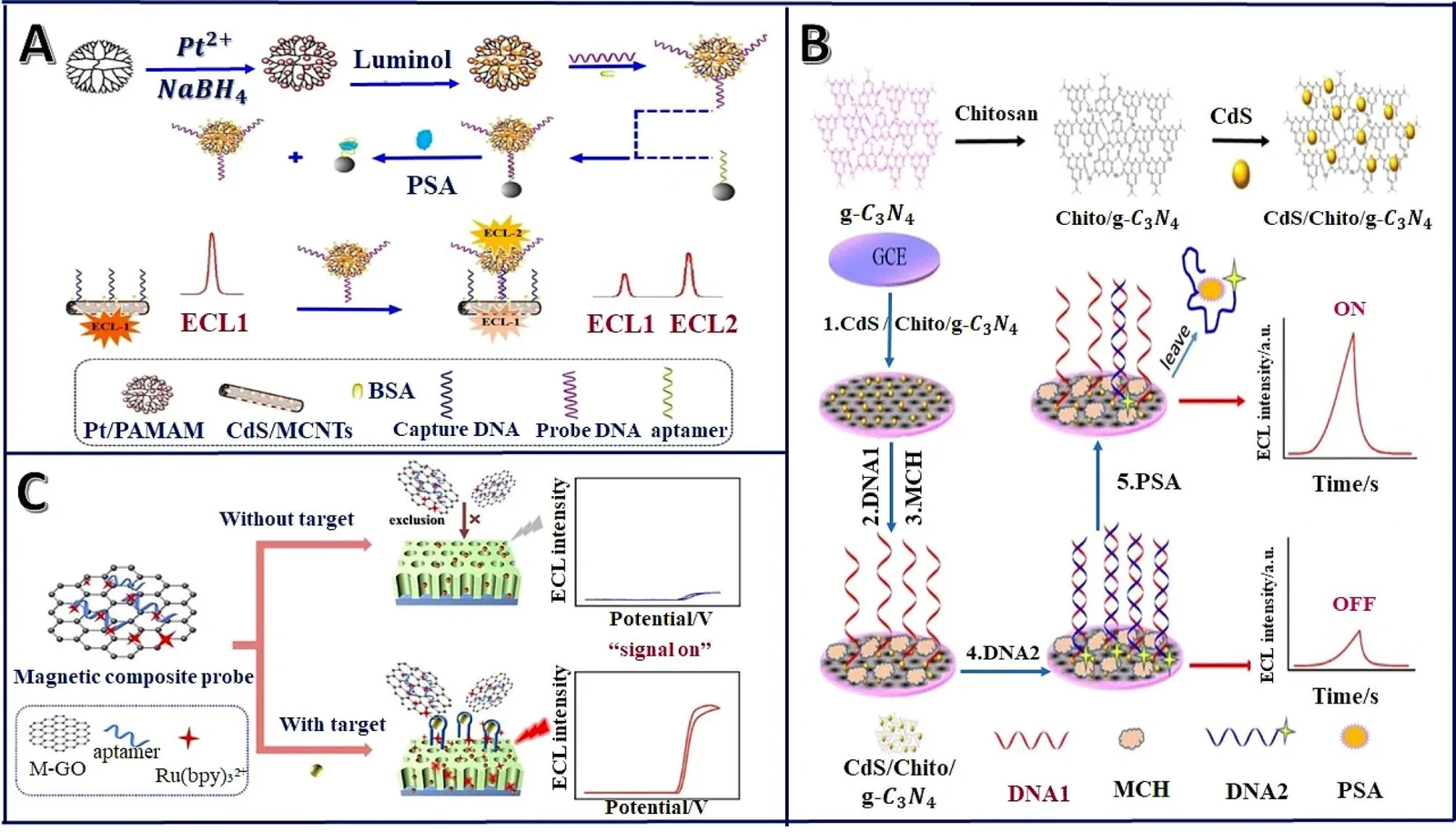
ECL aptasensors for PSA detection based on diverse nanomaterial designs: (A) An aptamer-based ECL platform using CdS/MCNT cathodic emitters and luminol-Pt/PAMAM anodic emitters. Reprinted with permission from Ref.^52^; (B) ECL aptasensor employing CdS/Chito/g-C₃N₄ nanocomposites modified with thiol- and ferrocene-labeled DNA probes, enabling signal recovery upon PSA binding. Reprinted with permission from Ref.^53^ (C) Magnetic graphene oxide-based ECL aptasensor for PSA and AFP detection using Ru(bpy)₃^2+^ and VMSF/ITO electrode. Reprinted with permission from Ref.^54^

 Huang et al. formed an ECL biosensor for PSA quantification by glassy carbon electrodes modified with CdS/Chitosan/g-C₃N₄ nanocomposites labeled with DNA bearing a thiol group and a ferrocene quencher.^53^ According to Figure 4B, in PSA absence, the ECL signal is extinguished due to vitality exchange between the emitter and the quencher. After the PSA is authorized, the DNA aptamer dissociates from the cathode, restoring the ECL intensity. A linearity of 1 pg mL^-1^ to 100ng mL^-1^, with a LOD of 0.14 pg mL^-1^ was found for PSA concentration in serum.^53^. Moreover, a sensitive ECL homogeneous aptasensor platform was developed using vertical mesoporous silica films (VMSF) and a magnetic graphene oxide (M-GO) probe containing Ru(bpy)₃^2+^ and a target-specific aptamer (Figure 4C).^54^ Following the aptamer-target binding, Ru(bpy)₃^2+^ is released and detected by a VMSF-modified ITO electrode, producing a strong signal with low background noise. The system successfully detected AFP and PSA with very low LOD (0.08 pg/mL and 9.6 pg/mL). It is low-cost, label-free, and does not require sample preparation or electrode alteration. Similarly, an ECL aptasensing platform based on hollow nanospheres constructed from Pt(II) complexes exhibiting aggregation-induced ECL (AIECL) behavior was developed by Chen et al.^55^ These platinum-based nanostructures, owing to their well-organized supramolecular assembly driven primarily by Pt-Pt interactions, served a dual function as highly efficient ECL emitters and signal-amplifying nanocatalysts. The resulting high-performance and ultrasensitive ECL biosensor achieved low LOD of 7.3 fg/mL over linear range of 10⁻⁵ to 10^2^ ng/mL in human serum.

####  Pro-gastrin-releasing peptide (31-98) (ProGRP31-98)

 ProGRP(31–98) is a stable fragment of the precursor to gastrin-releasing peptide (GRP), a neuropeptide involved in tumor growth and cell proliferation. It has been identified as a specific biomarker for small cell lung cancer (SCLC), displaying superior diagnostic accuracy and stability compared to neuron-specific enolase (NSE). Elevated ProGRP_31–98_ levels are strongly related to disease progression and tumor burden in SCLC patients. In the case of lung cancer, Cui and his colleagues found the determination of DNA aptamers for ProGRP_31-98_, an exceedingly solid and particular tumor marker for SCLC.^56^ According to Figure 5A, the aptamers were chosen utilizing the precise advancement of ligands by the exponential enhancement (SELEX) strategy.

**Figure 5:**
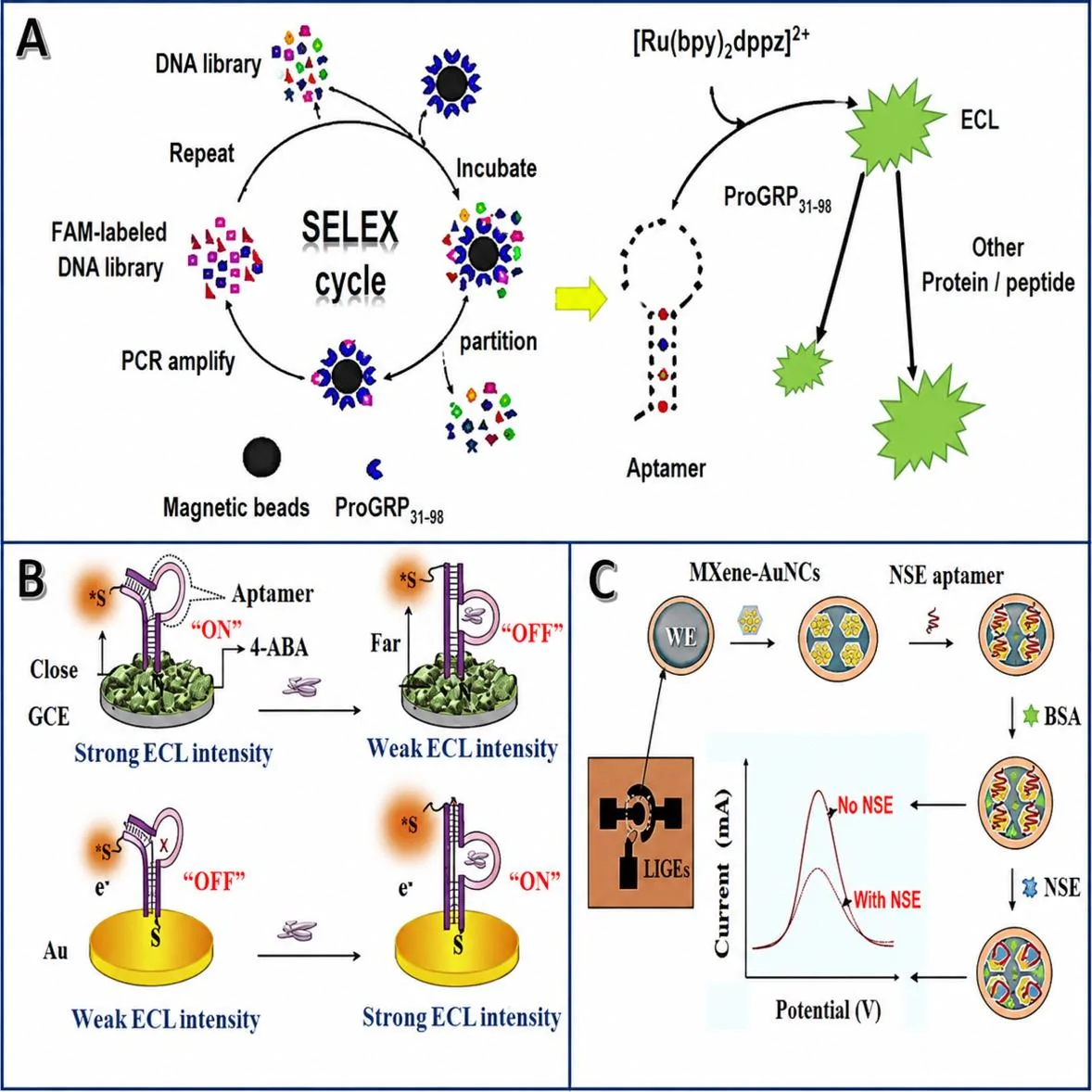
(A) Summary image of aptamer selection for the detection of the biomarker ProGRP31–98 for the diagnosis of SCLC by the exponential enrichment (SELEX) method. Reprinted with permission from Ref.^56^; (B) Graphical abstract of assay of NAP2 utilizing NBAT-Ru—a DNA-TWJ construct. Reprinted with permission from Ref.^57^; (C) Schematic of an MXene-AuNCs/LIGE-based ECL aptasensor for quantification of NSE in serum samples. Reprinted with permission from Ref.^58^

 To ensure the specific binding of selected DNA to ProGRP_31-98_, ECL measurement was used. The obtained aptamers exhibited high affinity toward ProGRP_31-98_, with a K_d_ value of 16 nM. and LOD of 17 nM.^56^

####  NAP2 

 NAP2, also known as CXCL7, is a chemokine primarily secreted by activated platelets and neutrophils. It plays an crucial role in tumor advancement and inflammation through its ability to stimulate leukocyte recruitment, angiogenesis, and cell proliferation. Elevated levels of NAP2 have been commonly connected with lung cancers. In this case, two innovative signal-off/on ECL deoxyribose sensors were established for straightforward, selective, sensitive, and early determination of NAP2 as the lung cancer biomarker. The recognition element in these sensors was NBAT-Ru, a Ru-labeled DNA three-way junction (DNA-TWJ). Signal-off and signal-on ECL deoxyribosensors based on NBAT-Ru showed high selectivity and sensitivity for NAP2 detection in plasma (Figure 5B). Both deoxyribosensors show exceptional selectivity and sensitivity, which are viably utilized for clinical plasma. These approaches represent distinct strategies based on the DNA-TWJ coordinates target-specific binding domain as the molecular recognition element, on one side, diverse immobilization strategies for biosensor creation, which hold critical guarantee for the determination of proteins, metal particles, microscopic organisms, and cells.^57^

####  Neuron-specific enolase (NSE) 

 As a glycolytic enzyme, NSE is found mainly within neurons and neuroendocrine tissues, serving as a well-established biomarker for SCLC. Elevated serum NSE levels correlate with tumor burden and disease progression, making it an important candidate for therapeutic monitoring and early detection. Due to its high specificity in neuroendocrine malignancies, ECL aptasensors developed for NSE detection have confirmed strong potential in offering sensitive, rapid, and non-invasive diagnostic platforms. In a study, Weng et al. considered the early quantification of lung cancer by NSE detection. As shown in Figure 5C, in this study, an aptasensor based on MXene-gold titanium carbide nanocomposites (MXene-AuNCs) and laser-induced graphene electrodes (LIGEs) was created by a polyamide film as a sensing system. To improve the electrical conductivity and immobilization capacity of aptamers, MXene-AuNCs were deposited on the electrode. MXene-AuNCs-Apt, as a labeling material, enhanced the signal. This aptasensor displayed a very low LOD of 0.13 pg/mL for NSE in serum samples, which offers a wide recognition range of 0.01 ng/mL-100 ng/mL in a specific and reproducible manner.^58^

###  Non-specific cancer biomarkers

####  Carcinoembryonic antigen (CEA) 

 CEA is a glycoprotein that is part of the immunoglobulin superfamily, possessing a molecular weight of 180 kDa.^75^ CEA is a widely-used tumor marker for several cancers, particularly in monitoring disease progression and metastasis.^59,76^ In Shi et al.’s work, a label-free system was considered and established for the identification of CEA in serum via cadmium sulfide-graphene (CdS-GR) nanocomposites. The Au–S bonds enabled the attachment of the thiolated aptamer on AuNPs, and the decrease in ECL signal after target binding was proportional to CEA concentration, achieving an LOD of 3.8 pg/mL.^59^ Liu et al. developed a spatially resolved sensor using CdS-C and luminol-AuNPs as cathodic and anodic emitters. ^60^ Ag-PAMAM-aptamer nanocomposites quenched the ECL signal, but in the existence of CEA, they detached, restoring the signal. LODs were achieved 0.20 pg/mL (anodic) and 0.39 pg/mL (cathodic) in human serum samples. Besides, Yang et al. developed a sandwich-type ECL aptasensor for CEA detection based on Au-CdS nanocomposites, using dual signal inhibition from hemin-graphene nanosheets and enzymatic biocatalytic precipitation.^61^ The dual quenching mechanism reduced ECL intensity upon target binding, enabling sensitive detection with a LOD of 0.28 pg/mL and a linear range of 0.8 pg/mL– 4 ng/mL. The design of this ECL aptasensor is straightforward and easy to operate, making it suitable for analyzing CEA levels in complex samples, with recovery rates ranging from 95.0% to 115.8%. In the study conducted by Liu et al., a label-free, inhibition-based ECL aptasensor was developed for the detection of CEA using an Au–LDH–C₃N₄ composite.^62^ In this system, the heterogeneous coupling between LDH and C₃N₄ improved the stability of ECL signal. The resulting system showed linearity in 0.001–20 ng/mL and an exceptionally low LOD of 0.3 pg/mL for CEA analysis. Overall, carbon nanomaterials and metal nanoparticles improve electron transfer and ECL intensity, while plasmonic and enzymatic amplification strategies provide much higher sensitivity.

####  Platelet-derived growth factor B chain (PDGF-BB) 

 Angiogenesis is a multifaceted process that plays a vital role in the growth of tumors and involves the activation of endothelial cells and interactions between multiple cell types. The platelet-derived growth factor (PDGF) family is one of the significant families of growth factors involved in increasing the migration and proliferation of pericytes and arterial smooth muscle cells. There exist four identified PDGF polypeptides: PDGF-A, PDGF-B, PDGF-C, and PDGF-D, which can create various heterodimers or homodimers. PDGF-BB is a homodimer of PDGF-B, exhibiting elevated expression levels in breast, colorectal cancer and promotes tumor lymphangiogenesis.^77,78^ Recently, investigators have established ECL-based aptasensors to monitor PDGF-BB.Lately, Chai et al. fabricated a platform by integrating N-(aminobutyl)-N-ethylisoluminol functionalized AuNPs (ABEI-AuNPs) with aptamers as nanoprobes for PDGF-BB, demonstrating high specificity and sensitivity. The aptamer capture probes, which were biotinylated, were first affixed to an electrode coated with streptavidin-functionalized gold nanoparticles (AuNPs).^63^ Also, Zhang et al. developed a sensitive ECL aptasensor for PDGF-BB using CdS QDs–PAMAM dendrimer probes functionalized with aptamers.^64^ A sandwich structure formed between two aptamers on MWCNT/chitosan-modified electrodes in the presence of PDGF-BB, enhancing ECL emission. The sensor showed a linear range of 0.5 pM–1 nM with an LOD of 0.13 pM, good recovery and low RSD. In a similar study showed by Gao et al., a β-cyclodextrin/graphitic carbon nitride (β-CD/g-C_3_N_4_) composite with high-performance ECL luminophores was used for the ultrasensitive and selective quantification of PDGF-BB.^65^ β-CD was non-covalently functionalized onto g-C_3_N_4_ to create an aptamer-based ECL platform. The system attained a very low LOD of 2.6 × 10^−13^ g/mL. β-CD increased the amount of adamantane-labeled DNA on the electrode, strengthening ECL quenching via photo-excited electron transfer and energy transfer between a ferrocene quencher and the g-C_3_N_4_ emitter.

####  Cancer antigen 125 (CA125)

 CA125is a protein cancer biomarker that is repeatedly found in higher concentrations in the blood of people with certain types of cancer, especially ovarian cancer. Additionally, CA125 concentrations may also be increased in several non-malignant conditions. In clinical practice, checking the serum concentration of CA125 can be used to identify and assess various conditions, including cancers, endometriosis, pelvic inflammatory disease, and liver disease. Therefore, it is important to provide methods for identifying CA125 with high accuracy and specificity.^66,79^ In a study by Zhang et al., researchers created a multi-amplified ECL aptasensor for the sensitive quantification of CA125 based on a toe-mediated strand displacement (TMSD) and tetrahedral DNA nanostructure (TDN) combined with AuNPs/Ru/ZIF signal probe.^66^ AuNPs/Ru/ZIF acted as the main ECL emitter and stabilized the TDN structure. Initially, ferrocene-labeled DNA probes suppressed the ECL signal (signal-off mode). Upon binding of the aptamer to CA125, a DNA primer triggered the TMSD process, releasing the quencher and restoring the ECL signal (signal-on mode). The recycling of the primer enhanced signal amplification, enabling a low LOD of 6 × 10⁻³ pg/mL. The aptasensor also demonstrated reliable identification of CA125.

 Unlike simple single-stranded DNA structures, the use of tetrahedral DNA nanostructures (TDNs) provides higher structural rigidity, enabling the probes to be organized on the electrode surface with improved geometric uniformity. This enhanced spatial arrangement directly contributes to better signal reproducibility and overall sensor performance.

####  Thrombin

 Thrombinis a serine protease that serves a crucial function in blood clotting and the coagulation cascade. It is implicated in various health conditions such as central nervous system damage, inflammatory responses, thromboembolic disease, alzheimer’s disease, and various types of cancer.^80^ Thrombin in the sub-endothelial matrix helps cells settle to new sites and promotes cancer spread through metastasis. Thrombin activity is increased in cancer due to increased thrombus formation and altered homeostasis associated with tumor; therefore, thrombin can be proposed as a biomarker in the diagnosis of all kinds of cancer.^81,82^ In a study conducted by Shan et al., an ECL aptasensor was developed for thrombin quantification based on a PTG-AuNPs modified electrode prepared by electropolymerizing thionine-doped graphene on GCE, followed by in situ AuNP deposition.^67^ Thrombin binding aptamer I was attached to the electrode as the capture probe, while Ru(bpy_)3_^2+^/AgNPs-doped silica core–shell nanocomposites labeled with aptamer II served as signal probes. The aptasensor exhibited a broad linearity of 2 fM–2 pM and a low LOD of 1 fM, demonstrating high sensitivity and potential for pharmaceutical and clinical applications. In additional study by Wang et al., the advancement of an ECL aptasensor for thrombin identification was achieved through the attachment of aptamers and the introduction of signal molecules.^68^ The researchers utilized GO to create a sensitive aptasensor. GO was physically adsorbed onto glass carbon or gold electrodes, thereafter covalent immobilization of an amino-tagged aptamer on electrode was achieved through amide bond formation involving the carboxyl groups present on GO. A functional oligonucleotide (FO) was considered to include an intermolecular duplex and a complementary strand for the intercalation of the ECL probe, Ru(phen)₃^2+^. The aptamer and its complementary strand hybridization allowed for the introduction of the Ru(phen)₃^2+^ probe, resulting in high ECL emission. When the aptamer bound to thrombin, it released the FO containing the Ru(phen)₃^2+^ probe, leading to a decrease in ECL emission that could be quantitatively measured to determine thrombin concentration. The sensor demonstrated a concentration-dependent response for thrombin with a LOD of 0.40 pM and 0.90 pM- 226 pM concentration. Additionally, GO not only facilitated aptamer immobilization but also enhanced sensitivity by preconcentrating tri-n-propylamine (TPrA) on its surface. This well-made label-free ECL aptasensor approach could be readily adapted for detecting other targets through the selection of appropriate aptamers. Also, A novel biosensor was developed for the quantification of thrombin utilizing QDs and ECL techniques.^69^ The process began with the immobilization of a thiol-terminated aptamer (probe I) containing 15 nucleotides onto a gold electrode. Thrombin was then introduced, forming bioaffinity complexes with the aptamer. Following this, a second aptamer (probe II), modified with 5′-biotin and consisting of 29 nucleotides, hybridized with the thrombin-aptamer complex. Streptavidin-modified QDs were subsequently attached to probe II through the biotin-avidin interaction. The ECL signal generated by the QDs was inversely related to combined thrombin amount, meaning that as thrombin concentration increased, the ECL intensity also amplified within the range of 0–20 μg/mL. The biosensor demonstrated exceptional stability and selectivity towards thrombin, making it a promising tool for detecting this target analyte.

## Challenges, trends, and future outlook

 Despite significant progress, several challenges still limit the translation of ECL aptasensors from laboratory settings to clinical and point-of-care applications. The most critical issue is the lack of reproducibility and standardization among laboratories, since variations in electrode modification, nanomaterial synthesis, and aptamer immobilization often lead to inconsistent analytical performance. Therefore, it will be essential to establish reference materials, standardized fabrication protocols, and unified evaluation criteria. In addition, multiplex analysis in complex biological matrices remains difficult because of luminophore spectral overlap, nonspecific adsorption, and insufficient aptamer stability under biological conditions.

 Emerging hybrid technologies, particularly CRISPR-based ECL systems, are beginning to address several of these limitations by introducing an additional layer of molecular recognition. CRISPR/Cas systems use programmable RNA-guided nucleases to recognize specific nucleic acid sequences with high precision. Their integration with ECL combines the molecular selectivity of CRISPR with the ultrahigh sensitivity of ECL, enabling highly specific detection of diverse biomarkers. Cas12a, an RNA-dependent endonuclease belonging to Class II CRISPR systems, is optimized for recognizing and cleaving double-stranded DNA through guidance by a sequence-complementary crRNA. Upon target recognition, activation of Cas12a induces cleavage of reporter probes, leading either to their release from the electrode or to modulation of an ECL-active signal.^83^ This mechanism amplifies the analytical response and significantly enhances detection sensitivity. Despite this, challenges related to system complexity, large-scale clinical validation, and reagent stability remain.^84^

 Integration with portable and point-of-care testing (POCT) systems represents both a major challenge and an emerging opportunity for ECL aptasensors. In the context of home healthcare and decentralized diagnostics, portable ECL devices must be rapid, cost-effective, user-friendly, and capable of operating with minimal sample volumes and reagents. Recent progress in miniaturized ECL platforms has enabled the development of portable systems incorporating alternative luminescence detection technologies, such as charge-coupled devices (CCDs) and complementary metal–oxide–semiconductor (CMOS) sensors, along with battery-powered or wireless energy supply units and integrated microfluidic chips.^85^

 In this context, microfluidic ECL systems represent an important advancement toward miniaturization and high-throughput analysis. These chip-based platforms are capable of manipulating and controlling extremely small sample volumes—typically in the microliter range—within microchannels, where a small electrode generates the ECL signal in situ. Owing to rapid mass transport in these systems, detection reactions occur faster, resulting in more uniform and stable signal generation. ^86,87^ For example, integration of microfluidic ECL technology with nanocomposite materials has demonstrated highly sensitive detection of cancer biomarkers such as HER2, with wide dynamic ranges and ultralow LOD.^88^ In addition, dual-mode microfluidic biosensors based on aptamer-functionalized nanostructures and advanced electrode architectures have shown promising performance in detecting extracellular vesicles in clinical samples. Generally, the incorporation of microfluidics with ECL significantly enhances assay sensitivity, speed, and portability. However, challenges associated with large-scale reproducibility and fabrication complexity remain.

 Smartphone-integrated digital platforms and microfluidic paper-based analytical devices (μPADs) with unique characteristics have emerged as particularly attractive solutions. The compatibility, biodegradability, and affordability of paper have led to the creation of various portable ECL aptasensors utilizing disposable μ-PADs.^89^ Additionally, introducing smartphones has enabled advanced image processing capabilities and high-resolution imaging, enhancing the ECL sensing process and simplifying the design of portable ECL devices. In some portable designs, the excitation current can be modulated via the smartphone audio jack, while ECL emission is directly captured by the built-in camera, allowing seamless integration of electrochemical excitation and optical readout within a single digital platform. In this regard, Yu’s group introduced an innovative ECL device featuring an array of four screen-printed carbon working electrodes capable of detecting various cancer cells.^90^ The carbon ink used in the screen-printed electrodes was modified with gold-platinum nanoparticles (Au-Pd NPs) and a selection of aptamers. The incorporation of Au-Pd NPs enhanced the ECL signal upon the aptamer’s recognition of cancer cells. This device exhibited remarkable analytical performance, repeatability, and stability in the detection of four distinct types of cancer cells. Furthermore, Kadimisetty et al. employed 3D printing technology to create a microfluidic device powered by a supercapacitor for the detection of proteins.^91^ This system relied on gravity flow for the delivery of reagents, the cleaning of samples, and utilized carbon electrodes that were printed manually. The sensors were capable of evaluating three PSA proteins, which serve as biomarkers for prostate cancer, in under 35 minutes. The LOD for the three proteins ranged from 300 to 500 fg mL^-1^.

 AI-assisted design is another emerging trend in ECL biosensing. Deep learning and machine learning algorithms have been applied to predict ECL responses, optimize sensor design, reduce background noise, and improve biomarker classification in complex biological samples. These approaches may also help address variability in nanomaterial synthesis, probe immobilization, and signal threshold determination. However, further validation is required before routine clinical implementation.^92-94^ Overall, the integration of advanced nanomaterials, CRISPR-based recognition, microfluidics, portable devices, and AI-assisted analysis is expected to accelerate the development of robust and clinically relevant ECL aptasensors.

## Conclusion and outlook

 Early detection of malignancies is crucial for reducing mortality rates associated with cancer. As such, innovative methods for identifying these diseases are continually being developed. Among these, electrochemiluminescence (ECL) has arisen as a great analytical platform for clinical diagnostics, particularly in the detection of cancer biomarkers in blood and other bodily fluids, offering lower error rates compared to traditional diagnostic methods. This review has specifically highlighted the recent progress in DNA aptamer-based ECL aptasensors, emphasizing their unique role in achieving high sensitivity and specificity in cancer biomarker detection. ECL offer various benefits over traditional antibodies, including low immunogenicity, high stability, ease of chemical synthesis, and the ability to undergo site-specific modifications. These features make DNA aptamers particularly well-suited for integration into ECL-based biosensors, where their highly selective target recognition directly translates into enhanced diagnostic accuracy. The ECL phenomenon, which merges electrochemistry with luminescence, allows for the generation of a detectable signal upon the binding of a target analyte to the DNA aptamer. A central theme of this review is the synergistic integration of advanced nanomaterials such as metal nanoparticles, carbon-based nanoparticles, and semiconductor nanoparticles into ECL aptasensors. These nanomaterials enhance sensor performance by providing increased surface area for improved aptamer immobilization, facilitating electron transfer through enhanced conductivity, and enabling signal amplification to achieve lower LOD. DNA aptamer-based ECL sensors have been successfully employed to identify b both specific and non-specific cancer biomarkers. Specific biomarkers, tied to particular malignancies, include breast cancer markers like HER2 and MUC1, prostate cancer marker PSA, and lung cancer marker NSE. For example, MUC1 was quantified via a DNA aptamer-AuNP ECL assay with excellent selectivity, while advanced dual-aptamer systems distinguish glycosylated versus total PSA for improved diagnostic specificity. Non-specific biomarkers, such as CEA, PDGF-BB, and thrombin, are found across multiple cancer types (and sometimes benign conditions), yet remain valuable in broader diagnostic panels. ECL aptasensors have achieved ultra-low LOD for CEA, and multiplex platforms simultaneously detecting CEA and NSE have demonstrated strong analytical performance. The integration of nanomaterials into ECL aptasensors has propelled these devices to the forefront of biosensing technology, enabling more accurate and timely cancer detection. Continued research and innovation are essential to further optimize aptamer design, enhance biosensor robustness, and facilitate translation into clinical applications. As advanced biosensor technologies, particularly ECL, continue to mature and integrate into healthcare practices, they hold immense potential to transform cancer diagnosis and monitoring, paving the way for more effective and personalized treatment approaches.

## Competing Interests

 The authors declare that they have no known competing financial interests or personal relationships that could have appeared to influence the work reported in this paper.

## Data Availability Statement

 The data that has been used is confidential.

## Ethical Approval

 Not applicable.
